# Sleep‐disordered breathing in heart failure patients with different etiologies

**DOI:** 10.1002/clc.23840

**Published:** 2022-05-10

**Authors:** Tao Wang, Fu‐Chao Yu, Qin Wei, Xuan Xu, Liang Xie, Ning Ding, Jia‐Yi Tong

**Affiliations:** ^1^ Department of Cardiology, Zhongda Hospital, School of medicine Southeast University Nanjing China; ^2^ Department of Respiratory and Critical Care Medicine The First Affiliated Hospital of Nanjing Medical University Nanjing China

**Keywords:** etiologies, heart failure, prevalence, sleep‐disordered breathing

## Abstract

**Background:**

The prevalence of sleep‐disordered breathing (SDB) is closely related to the severity of heart failure (HF), and the severity of HF is different in patients with HF of different etiologies.

**Hypothesis**: This study aimed to explore the prevalence of SDB in patients with HFof different etiologies.

**Methods:**

Hospitalized HF patients were consecutively enrolled. All patients underwent portable overnight cardiorespiratory polygraphy. Patients were divided into five groups according to the etiology of HF: ischemic, hypertensive, myocardial, valvular, and arrhythmic. The prevalence of SDB and clinical data was compared among the five groups.

**Results:**

In total, 248 patients were enrolled in this study. The prevalence of SDB in HF was 70.6%, with the prevalence of obstructive sleep apnea (OSA) at 47.6% and central sleep apnea (CSA) at 23.0%. Patients were divided into five groups: ischemic, hypertensive, myocardial, valvular, and arrhythmic. The prevalence of SDB among the five groups was 75.3%, 81.4%, 77.8%, 51.9%, and 58.5% (*p* = .014), respectively. The prevalence of OSA among the five groups was 42.7%, 72.1%, 36.1%, 37.0%, and 49.1% (*p* = .009), whereas the CSA was 32.6%, 9.3%, 41.7%, 14.8%, and 9.4% (*p* < .001), respectively.

**Conclusions:**

SDB is common in HF patients. The prevalence and types of SDB varied in HF with different etiologies, which may be related to the different severities of HF. SDB was highly prevalent in patients with ischemic, hypertensive, and myocardial HF. Hypertensive HF patients were mainly complicated with OSA, while myocardial HF patients were mainly complicated with CSA. Both conditions were highly prevalent in ischemic HF patients. The prevalence of SDB was relatively low in valvular and arrhythmic HF patients, and OSA was the main type.

## 1 BACKGROUND

Sleep‐disordered breathing (SDB), which is mainly divided into obstructive sleep apnea (OSA) and central sleep apnea (CSA), has become a worldwide public health problem with a high prevalence in cardiovascular diseases, especially in patients with heart failure (HF), with a prevalence of approximately 50%–70%.^1^, ^2^ OSA patients with intermittent hypoxia cause sympathetic activation, endothelial dysfunction, hypercoagulability, inflammation, oxidative stress, metabolic dysregulation, and so forth, leading to various cardiovascular diseases and ultimately HF.^3^ As HF progresses, patients develop severe water and sodium retention and increased venous reflux at night when adopting the supine position leading to fluid retention around upper airway tissue, which causes the upper airway to narrow, making it more prone to collapse and potentially inducing OSA.^4^, ^5^ CSA is generally considered to occur as a result of HF, and is considered to be a potential marker of advanced HF. The prevalence of CSA increases with the deterioration of New York Heart Association (NYHA) class,^6^ and the occurrence and severity of CSA in HF patients reflect HF severity.^7^ When HF is aggravated, severe congestion in the lungs leads to hyperventilation, resulting in a decrease in PaCO_2_, which, in turn, induces CSA.^8^ These findings suggest the prevalence and types of SDB are affected by the severity of HF, particularly CSA. Different etiologies of HF occur due to different pathogenic mechanisms, the degree of myocardial damage is different between these etiologies, meaning the severity of HF varies, and the prevalence and types of SDB may also vary. The purpose of this study is to investigate the prevalence and characteristics of SDB in patients with HF of different etiologies.

## 2 METHODS

### 2.1 Subjects

Patients with HF who were admitted to the Department of Cardiology, Zhongda Hospital affiliated with Southeast University between January 2021 and October 2021 were enrolled.

Inclusion criteria: 1) adherence to the diagnostic criteria of the 2016 European Society of Cardiology guidelines for HF; 2) chronic HF or stable acute HF (patients with acute HF were enrolled after their condition stabilized), defined as NYHA functional Class II–IV; 3) the etiologies of HF were ischemic heart disease, hypertension, dilated cardiomyopathy (DCM), valvular disease or arrhythmias; 4) age: 18–85 years old; and 5) provided signed informed consent.

Exclusion criteria:1) heart surgery performed within 6 months; 2) acute myocardial infarction; 3) severe renal insufficiency (estimated glomerular filtration rate 30 ml/min/1.73 m^2^) or renal transplantation; 4) active liver disease or liver dysfunction (aspartate transaminase or alanine transaminase ≥3 times the normal upper limit); 5) malignant tumor; 6) active infection or severe blood disease; 7) history of chronic obstructive pulmonary disease, asthma, or other serious lung diseases; 8) history of stroke, peripheral or central neurological abnormalities, or cerebrovascular diseases; 9）use of morphine and its analogs, sedatives, and theophylline drugs; and 10) mental disorder.

The etiology of HF was defined based on past medical history and echocardiogram results. Ischemic HF is caused by ischemic heart disease, mainly coronary heart disease (CHD). Hypertensive HF is caused by hypertension. In our study, myocardial HF was caused by DCM. Valvular HF is caused by valvular heart disease and arrhythmic HF is caused by arrhythmias, such as atrial fibrillation, atrial flutter, atrial tachycardia, supraventricular tachycardia, atrioventricular block, and sinus node dysfunction.

### 2.2 Clinical assessment

The clinical data of patients were recorded, including age, sex, anthropometric data (height, weight, body mass index [BMI], neck circumference, waist circumference, basal blood pressure), smoking history, drinking history, snoring history, and any comorbidities.

### 2.3 Cardiac function assessment

NYHA classification was assessed immediately after the patients were enrolled. The 6‐min walk test (6MWT) was performed 3 days after hospital admission according to the guidelines issued by the American Thoracic Society.

### 2.4 Questionnaire

The Epworth sleepiness scale was performed immediately after the patients were enrolled according to the guidelines issued by the American Sleep Disorders Association and Sleep Research Society.

### 2.5 Blood parameters

Blood samples were collected during fasting in the morning after admission to test the N‐terminal brain natriuretic peptide (NT‐proBNP).

### 2.6 Echocardiography

Echocardiography was performed using ViVid E95 (GE Healthcare) and evaluated by experienced cardiac sonographers. The left ventricular ejection fraction (LVEF), left atrial diameter (LA), left ventricular end‐diastolic diameter (LV), right atrial diameter (RA), and right ventricular diastolic diameter (RV) were recorded.

### 2.7 Cardiorespiratory polygraphy

All patients underwent portable overnight polygraphy (ApneaLink Plus sleep monitor; ResMed Co.) during the hospitalization and none of the patients received oxygen therapy at night. A nasal cannula was placed in the nasal cavity, respiratory airflow was measured using disposable airflow sensors, respiratory movement in chest walls were monitored by strain sensors, and SpO_2_ was continuously monitored by a pulse oximeter. A specialist analyzed the sleep reports using the manual relevant to the machine. Sleep apnea (SA) was defined as the cessation of airflow through the nose and mouth for >10 s during sleep. Obstructive apnea (OA) was defined as the cessation of nasal and oral airflow for >10 s, accompanied by chest movement. Central apnea (CA) was defined as the cessation of nasal and oral airflow for >10 s without chest movement. Hypopnea was defined as a reduction in respiratory airflow intensity (amplitude) of >50% from the basal level during sleep, accompanied by a ≥4% decrease in blood oxygen saturation (SaO_2_) from the basal level, lasting for >10 s. SDB was defined as more than 30 recurrent episodes of apnea and hypopnea per 7 h of sleep per night, or the apnea‐hypopnea index (AHI), defined as apnea plus hypopnea, with an average of five or more episodes per hour of sleep per night. SDB was divided into OSA (OA events >50% of total events) and CSA (CA events >50% of total events). AHI ranges of 5–14 were considered to denote mild SDB, 15–29 moderate SDB, and ≥30 severe SDB.

### 2.8 Statistical analysis

SPSS statistical software (version 22.0; IBM Corp.) was used for the statistical analyses. The normal continuous data were expressed as mean ± standard deviation, and comparison between groups was performed by one‐way analysis of variance, and Bonferroni or Tamhane's test was performed for post hoc multiple comparisons. Median (quartiles) was used for non‐normal continuous data, a nonparametric test was used for the intergroup comparison, and the Kruskal–Wallis test was performed for the post hoc test. Categorical variables were presented as frequencies and percentages, and the comparison between the groups was performed by the *χ* ^2^ test or Fisher's exact test. *p* < .05 was considered to indicate statistical significance.

## 3 RESULTS

### 3.1 Epidemiology

**Figure 1:**
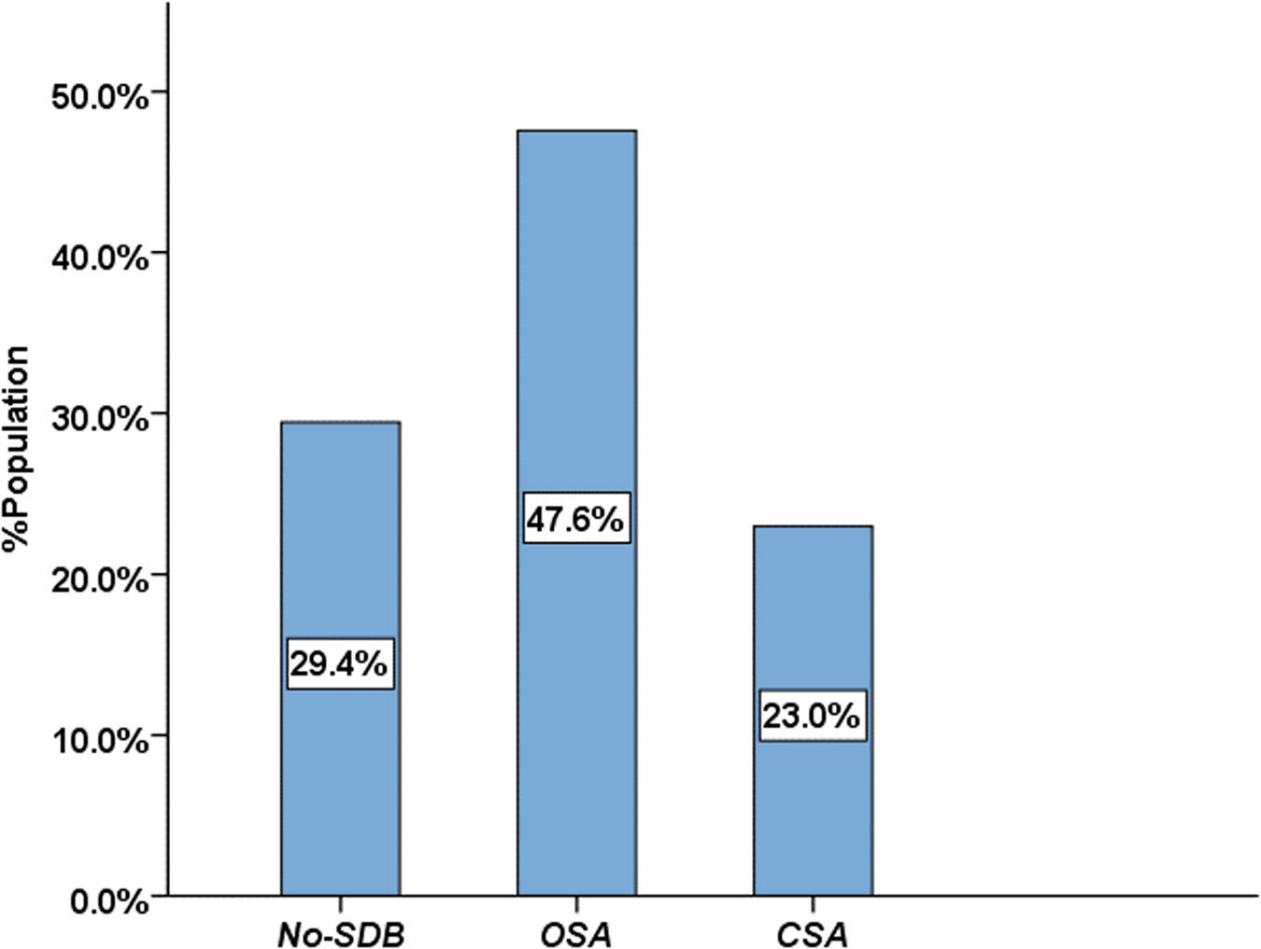
Prevalence of SDB (OSA and CSA) in HF patients independent of etiologies. CSA, central sleep apnea; HF, heart failure; OSA, obstructive sleep apnea; SDB, sleep‐disordered breathing.

**Figure 2:**
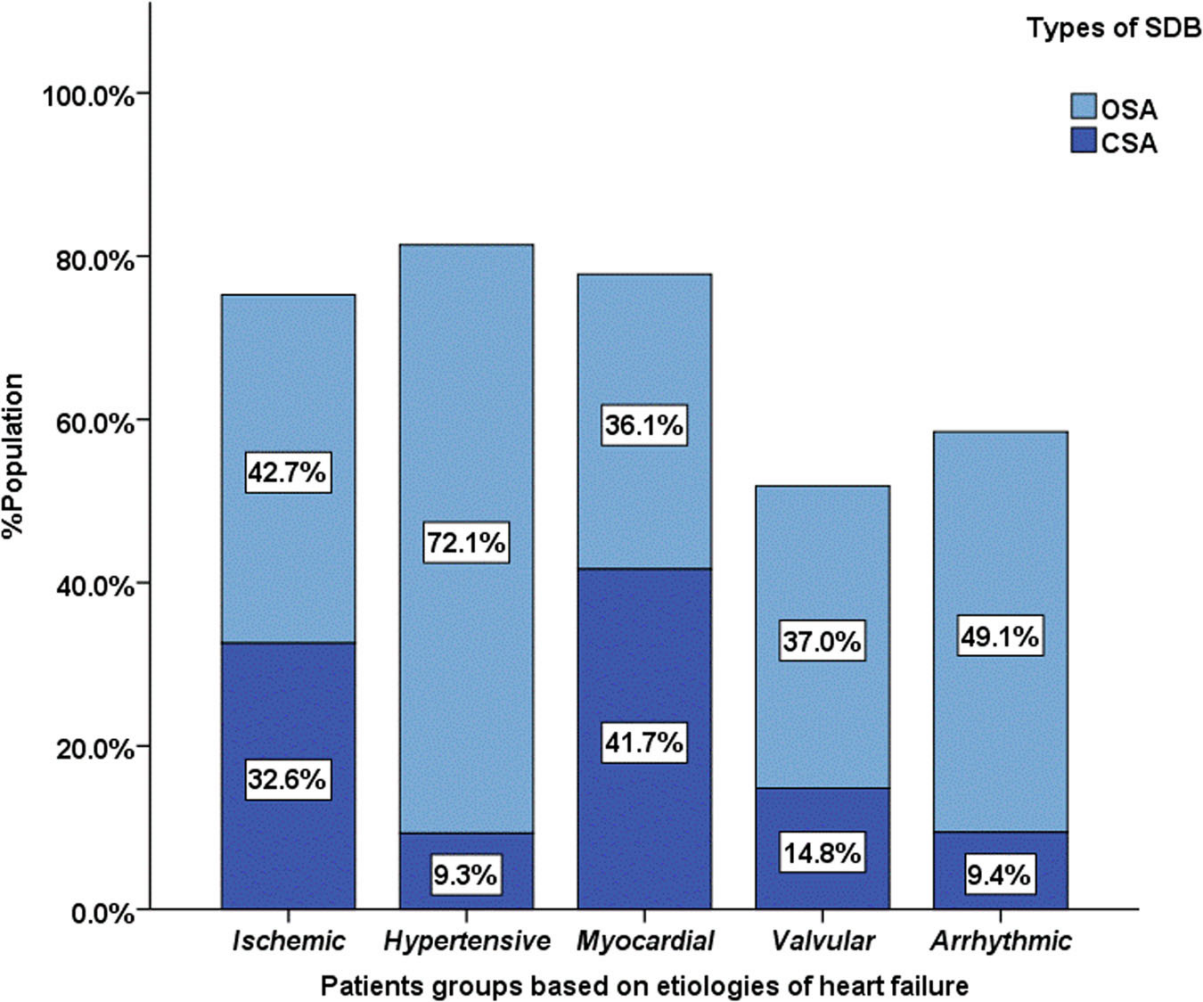
Prevalence of SDB in patients with HF of different etiologies. CSA, central sleep apnea; HF, heart failure; OSA, obstructive sleep apnea; SDB, sleep‐disordered breathing.

A total of 248 patients with HF were enrolled in the study, an average age of 70.4 ± 12.4 years old, and 132 were men (53.2%). The prevalence of SDB in HF was 70.6%, while that of OSA was 47.6% and CSA was 23.0% (Figure 1). Patients were divided into five groups according to the etiology of HF: ischemic, hypertensive, myocardial, valvular, and arrhythmic. The prevalence of SDB among the five groups was 75.3%, 81.4%, 77.8%, 51.9%, and 58.5% (*p* = .014), respectively. The prevalence of OSA among the five groups was 42.7%, 72.1%, 36.1%, 37.0%, and 49.1% (*p* = .009), whereas the prevalence of CSA among the five groups was 32.6%, 9.3%, 41.7%, 14.8%, and 9.4% (*p* < .001), respectively (Figure 2).

### 3.2 Clinical characteristics

Table 1 shows that the proportion of smokers in the ischemic group was higher than that in the other four groups (*p* = .040). The basal blood pressure, waist circumference, and neck circumference in the hypertensive group were higher than those in the other four groups (*p* < .05). The age in the myocardial group was younger than that of the other four groups (*p* = .01). NT‐proBNP and the proportion of NYHA Class III/IV in the ischemic and myocardial groups were higher than those in the other three groups (*p* < .05). The proportion of snoring in the ischemic, hypertensive and myocardial groups was higher than that in the other two groups (*p* < .05).

**Table 1:** Patient characteristics, anthropometric measures, and cardiac function assessment among HF patients with different etiologies.

	**Ischemic**	**Hypertensive**	**Myocardial**	**Valvular**	**Arrhythmic**	
**Variables**	89 (35.9%)	43 (17.3%)	36 (14.5%)	27 (10.9%)	53 (21.4%)	***p***
Age (y)	73.0 (66.0–81.5)	75.0 (68.0–82.0)	67.0 (56.0–75.0)*	73.0 (62.0–82.0)	70.0 (61.5–79.0)	.010
Sex(male), *n* (%)	52 (58.4%)	21 (48.8%)	21 (58.3%)	8 (29.6%)	5 (56.6%)	.093
BMI (kg/m^2^)	24.0 (21.9–26.7)	25.5 (22.9–28.7)	24.3 (21.2–27.2)	23.9 (20.1–25.8)	24.8 (22.2–27.8)	.140
Waist circumference (cm)	92.0 (85.5–98.0)	97.0 (87.5–103.0)	90.8 (79.6–98.8)*	85.0 (80.0–91.0)	92.5 (84.0–99.0)	.005
Neck circumference (cm)	37.5 (34.3–40.0)	38.5 (35.0–40.5)	36.3 (33.5–39.8)	34.5 (32.0–37.5)*, **	36.5 (34.3–39.8)	.006
SBP (mmHg)	130.7 ± 25.2	149.6 ± 24.3**	123.8 ± 18.6*	123.9 ± 26.0*	127.6 ± 18.1*	＜.001
DBP (mmHg)	75.0 (65.5–81.0)	80.0 (72.0–91.0)	76.0 (65.3–84.3)	67.0 (53.0–78.0)*	73.0 (67.5–88.5)	.009
Smoking, *n* (%)	36 (40.4%)	14 (32.6%)	10 (27.8%)	3 (11.1%)**	13 (24.5%)	.040
Drinking, *n* (%)	24 (27.0%)	8 (18.6%)	9 (25.0%)	3 (11.1%)	14 (26.4%)	.437
Snoring, *n* (%)	59 (66.3%)	33 (76.7%)	28 (77.8%)	7 (25.9%)*, **, ***	10 (18.9%)*, **, ***	＜.001
ESS score	6.0 (2.0–11.0)	5.0 (2.0–9.0)	7.0 (3.0–12.0)	5.0 (3.0–9.0)	5.0 (2.0–7.0)	.120
NT‐proBNP (pg/ml)	1870 (1272–2700)	855 (418–1840)**	2130 (1010–6750)*	1260 (643–4170)	1580 (849–2545)	＜.001
6MWT (m)	285 ± 123	313 ± 100	256 ± 85	304 ± 77	316 ± 116	.068
NYHA class, *n* (%)						
II	26 (29.2%)	29 (67.4%)**	9 (25.0%)*	17 (63.0%)**, ***	30 (56.6%)**, ***	＜.001
III + IV	63 (70.8%)	14 (32.6%)**	27 (75.0%)*	10 (37.0%)**, ***	23 (43.4%)**, ***	＜.001

*Note*: Data are presented as the percentage of the cohort, mean ± standard deviation, or median (quartiles).

Abbreviations: 6MWT, 6‐min walk test; BMI, body mass index; DBP, diastolic blood pressure; ESS, Epworth sleepiness scale score; HF, heart failure; NT‐proBNP, N‐terminal brain natriuretic peptide; NYHA, New York Heart Association functional class; SBP, systolic blood pressure.

* *p* < .05, myocardial/valvular/arrhythmic groups versus hypertensive group.

** *p* < .05, hypertensive/myocardial/valvular/arrhythmic groups versus ischemic group.

*** *p* < .05, valvular/arrhythmic groups versus myocardial group.

### 3.3 Echocardiographic parameters

As shown in Table 2, the LA, LV, RA, and RV were significantly higher in the myocardial group than those in the other four groups (*p* < .05). The LVEF values in the myocardial group were the lowest, followed by those in the ischemic group, while those in the other three groups were higher (*p* < .001).

### 3.4 Sleep study data

A comparison of sleep data among the five groups (Table 3) showed that AHI, longest time of hypopnea, and proportion of Cheyne–Stokes respiration (CSR) in the ischemic, hypertensive, and myocardial groups were higher than those in the valvular and arrhythmic groups (*p* < .05). The proportion of moderate‐to‐severe SDB in the ischemic, hypertensive, and myocardial groups was higher than that in the valvular and arrhythmic groups (*p* < .05).

**Table 2:** Echocardiographic parameters in different groups of heart failure.

	Ischemic	Hypertensive	Myocardial	Valvular	Arrhythmic	
Variables	89 (35.9%)	43 (17.3%)	36 (14.5%)	27 (10.9%)	53 (21.4%)	*P*
LA (cm)	4.3 ± 0.7	4.2 ± 0.7	5.2 ± 0.6*, **	4.6 ± 0.9***	4.7 ± 1.0***	＜.001
LV (cm)	5.0 ± 1.0	4.9 ± 0.7	5.8 ± 1.1*, **	5.3 ± 0.9	4.9 ± 0.7***	＜.001
RA (cm)	4.0 ± 0.7	4.0 ± 0.5	4.8 ± 0.5*, **	4.6 ± 1.0	4.4 ± 0.8	＜.001
RV (cm)	2.5 ± 0.4	2.5 ± 0.3	2.8 ± 0.3*, **	2.6 ± 0.6	2.5 ± 0.4***	＜.001
LVEF	0.58 (0.43–0.68)	0.69 (0.61–0.75)*	0.43 (0.31–0.52)**	0.64 (0.55–0.70)***	0.61 (0.52–0.69)***	＜.001

*Note*: Data are presented as mean ± standard deviation, or median (quartiles).

Abbreviations: LA, left atrial diameter; LV, left ventricular end‐diastolic diameter; LVEF, left ventricular ejection fraction; RA, right atrial diameter; RV, right ventricular diastolic diameter.

* *p* < .05, hypertensive/myocardial/valvular/arrhythmic groups versus ischemic group.

** *p* < .05, myocardial/valvular/arrhythmic groups versus hypertensive group.

*** *p* < .05, valvular/arrhythmic groups versus myocardial group.

**Table 3:** Sleep data in different groups of heart failure.

	Ischemic	Hypertensive	Myocardial	Valvular	Arrhythmic	
Variables	89 (35.9%)	43 (17.3%)	36 (14.5%)	27 (10.9%)	53 (21.4%)	*P*
AHI (per h)	18.3 (5.0–31.4)	12.8 (6.1–28.0)	20.3 (9.3–34.5)	6.6 (1.7–22.5)*	6.9 (3.6–20.5)	.009
Average SaO_2_ (%)	93.3 ± 2.5	93.1 ± 1.9	92.9 ± 2.1	93.5 ± 1.7	93.7 ± 2.6	.441
Lowest SaO_2_ (%)	81.0 75.0–85.5)	80.0 (76.0–84.0)	81.0 (75.3–84.0)	77.0 (74.0–84.0)	80.0 (72.5–82.5)	.755
Baseline SaO_2_ (%)	96.0 (94.0–98.0)	95.0 (93.0–97.0)	96.0 (94.3–97.0)	96.0 (94.0–98.0)	96.0 (95.0–98.0)	.101
Longest time of hypopnea (s)	80.8 ± 26.4	83.1 ± 18.5	87.6 ± 18.9	63.6 ± 30.9*, **, ***	75.9 ± 28.2	.003
Longest time of apnea (s)	44.4 ± 22.0	40.0 ± 17.2	47.6 ± 23.1	36.4 ± 22.8	40.2 ± 20.1	.182
CSR, *n* (%)	49 (55.1%)	19 (44.2%)	22 (61.1%)	7 (25.9%)*, **	15 (28.3%)*, **	.002
With SDB, *n* (%)	67 (75.3%)	35 (81.4%)	28 (77.8%)	14 (51.9%)***	31 (58.5%)***	.014
Type of SDB, *n *(%)						
OSA	38 (42.7%)	31 (72.1%)**	13 (36.1%)***	10 (37.0%)***	26 (49.1%)	.009
CSA	29 (32.6%)	4 (9.3%)**	15 (41.7%)***	4 (14.8%)	5 (9.4%)*, **	＜.001
Severity of SDB, *n *(%)						
Mild	20 (22.5%)	14 (32.6%)	7 (19.4%)	6 (22.2%)	15 (28.3%)	.622
Moderate‐to‐severe	47 (52.8%)	21 (48.8%)	21 (58.3%)	8 (29.6%)*	16 (30.2%)	.016

*Note*: Data are presented as the percentage of the cohort, mean ± standard deviation, or median (quartiles).

Abbreviations: AHI, apnea‐hypopnea index; CSA, central sleep apnea; CSR, Cheyne–Stokes respiration; OSA, obstructive sleep apnea; SaO_2_, oxygen saturation; SDB, sleep‐disordered breathing.

* *p* < .05, valvular/arrhythmic groups versus myocardial group.

** *p* < .05, hypertensive/myocardial/valvular/arrhythmic groups versus ischemic group.

*** *p* < .05, myocardial/valvular/arrhythmic groups versus hypertensive group.

## 4 DISCUSSION

In this study, we showed that SDB was highly prevalent among patients with HF, reaching a prevalence of 70.6%, comprising predominantly OSA (47.6%) and CSA (23.0%). The prevalence of SDB varied with HF caused by different etiologies. In our study, we found that 42.7% of the patients with ischemic HF had OSA, whereas 32.6% had CSA. Similarly, Salama et al.^9^ enrolled 100 HF patients to investigate the prevalence of SDB in ischemic heart disease, hypertensive heart disease, and cardiomyopathy and found that the prevalence of OSA and CSA in patients with ischemic heart disease was 47.9% and 37.5%, respectively. Prinz et al.^10^ conducted cardiorespiratory polygraphy in 275 patients with ischemic heart disease and found that 51% of patients had OSA and 23% had CSA. Our results are consistent with those of previous studies, suggesting a high prevalence of SDB in ischemic HF. Compared with valvular HF and arrhythmic HF, the high prevalence of SDB in patients with ischemic HF was mainly due to the increase in CSA, whereas the prevalence of OSA was not significantly increased. In addition, the severity of SDB in the ischemic HF group was significantly higher than that in the other two groups, with a higher average AHI, higher proportion of CSR, and moderate‐to‐severe SDB. To this end, we further compared the clinical data between the three groups and found that the cardiac function indicators of the ischemic group, such as NT‐proBNP, and the proportion of NYHA Class III/IV, were higher than those in the above two groups, while LVEF, 6MWT distance was lower than the above two groups, suggesting that the severity of HF was higher in patients with ischemic HF. Several prior studies have shown that the severity of HF is closely related to the prevalence and severity of SDB and CSA, and the occurrence and severity of CSA also reflect potential cardiac dysfunction.^2^, ^7^, ^11^ CSA is often associated with elevated BNP, low LVEF, and high NYHA class,^12^, ^13^, ^14^ and CSA prevalence increases as NYHA class deteriorates.^6^ The high severity of ischemic HF may be caused by myocardial cell necrosis after long‐term ischemia, hypoxia, or myocardial infarction, which leads to a severe decline in myocardial systolic function and severe water and sodium retention, resulting in a high prevalence of SDB and CSA. We also found that the ischemic group had the highest rate of smoking in our study, which is a risk factor for CHD.^15^ This finding is consistent with the characteristics of patients with ischemic HF.

Myocardial HF (DCM) was more severe than ischemic HF in our study, with the lowest LVEF, the highest NT‐proBNP, the highest proportion of NYHA Class III/IV, the largest atrioventricular diameter, and the shortest 6MWT distance. Our study further revealed that CSA was a prevalent disorder among DCM patients, with an estimated prevalence of 41.7%, compared to 36.1% of patients with OSA. The proportion of CSA was significantly higher than that of the other four groups, and the severity of SDB was also the highest, which manifested in the highest AHI, longest hypopnea time, the highest CSR ratio, and the highest proportion of moderate‐to‐severe SDB. This was due to the fact that the whole heart of DCM patients was enlarged and the myocardial systolic function was seriously decreased, meaning the cardiac function was seriously impaired and the retention of water and sodium was aggravated, leading to hyperventilation and decreased PaCO_2_, which significantly increased the prevalence of CSA and SDB and increased the severity of SDB.^8^ Similarly, Banno et al.^16^ observed the prevalence of SDB in 35 patients with DCM and found that the prevalence of CSA and OSA were 50% and 30%, respectively. In another study, Javaheri et al.^17^ enrolled 81 patients with HF and found that the prevalence of CSA in DCM was 45%. In addition, Salama et al.^9^ also found a high prevalence of CSA in DCM (up to 50%), while that of OSA was 39.3%. Our results are consistent with those of these prior studies. In addition, we found that this group of patients was the youngest, which was consistent with the characteristics of early onset of DCM.

In this study, the severity of HF in the valvular group was lower, with a higher LVEF, lower NT‐proBNP, longer 6MWT distance, and a higher proportion of NYHA Class II. The lower severity of HF in the valvular group resulted in a relatively lower prevalence of SDB (51.9%), with OSA and CSA accounting for 37.0% and 14.8%, CSA was mainly reduced. A previous study reported that the prevalence of SDB in patients with rheumatic valvular heart disease was 38.8%, among which OSA accounted for 16.2% and CSA for 22.7%.^18^ Lombardi et al.^19^ found that the prevalence of OSA and CSA was 35.3% and 29.4% in patients with valvular heart disease. The prevalence of CSA in our study was a little lower than that of the previous studies, which may be due to the fact that some patients included in our study had undergone valve replacement surgery. Several studies have shown that after valve replacement, cardiac function is improved, and CSA is reduced.^20^, ^21^ This led to a lower prevalence of CSA in our study than in previous studies.

Similarly, the severity of HF was relatively low in patients in the arrhythmic group in this study. We found that the prevalence of SDB in patients with arrhythmic HF was 58.5%, OSA was also dominant, accounting for 49.1%, and CSA accounted for 9.4%. However, the prevalence of SDB in arrhythmic HF has not been previously reported. This study included patients with arrhythmias, such as atrial fibrillation, atrial flutter, atrial tachycardia, supraventricular tachycardia, atrioventricular block, and sinus node dysfunction. Owing to abnormal myocardial systolic function or bradycardia, insufficient cardiac output in these patients leads to myocardial ischemia, hypoxia, and induces HF. However, with arrhythmias under control, patients in this group generally experienced milder HF. Thus, the prevalence of CSA was lower and the severity of SDB was less severe.

Although patients with hypertensive HF in this study also had a low degree of HF, which manifested as the highest LVEF, lowest NT‐proBNP, and the highest proportion of NYHA Class II, the prevalence of SDB in hypertensive HF reached up to 81.4%. We further found that although the prevalence of SDB was high in the hypertensive group, OSA was dominant, accounting for 72.1%, whereas CSA accounted for only 9.3%. This finding is closely related to the characteristics of hypertension. Most patients with hypertension are obese. In this study, the waist and neck circumferences in the hypertensive group were significantly higher than those in other patients, and the BMI was also slightly higher than that in other groups. Increases in waist circumference, neck circumference, and BMI were independent risk factors for OSA, and the severity of OSA was also closely related to obesity.^22^, ^23^, ^24^ Additionally, patients with hypertensive HF generally had preserved ejection fraction,^25^ which manifests as diastolic dysfunction.^23^ The degree of HF was relatively low. These resulted in a high prevalence of OSA and a low prevalence of CSA. The results of our study were similar to those of Salama et al.,^9^ who found the prevalence of OSA and CSA in hypertensive HF patients was 79.2% and 20.8%, respectively. Similarly, Logan et al.^26^ identified the prevalence of SDB in patients with hypertensive heart disease and found that OSA was as high as 83%. In addition, Lombardi et al.^19^ observed the prevalence of SDB in 369 patients with HF and found that the prevalence of OSA in hypertensive heart disease was as high as 81.8%. Both our and previous studies have suggested that the prevalence of SDB in patients with hypertensive HF is high and that OSA is dominant.

Interestingly, the prevalence of OSA in ischemic, myocardial, valvular, and arrhythmic HF groups were not significantly different. Studies have reported that fluid retention is aggravated with the aggravation of HF, and fluid transfer to the upper airway tissue at night aggravates upper airway obstruction, which can further trigger and aggravate OSA.^4^, ^5^ However, there was no difference in the prevalence of OSA among the groups in our study. The possible reasons were as follows: 1) Obesity was an important risk factor for OSA, while there was no significant difference in BMI, neck circumference, and waist circumference among the four groups. 2) As HF progresses, OA events and CA events both increase, but CA events show greater increments. Patients with both OA and CA were classified according to the dominating respiratory sleep pattern by a sleep monitor. Therefore, these patients were divided into the CSA group due to the greater increment of CA.

## 5 LIMITATIONS

First, although overnight polygraphy is considered the “gold standard” for the diagnosis of SDB, it also has several limitations: (1) Patient AHI differs between nights; (2) the test usually requires the participants to be in a supine position regardless of the subject's sleep habits; and (3) some patients are prone to sleep difficulties in an unfamiliar environment. These factors may influence the actual value of the AHI. Second, this was a single‐center study with small sample size, and its conclusions need to be further verified by multicenter studies with large samples.

## 6 CONCLUSION

SDB is common in HF patients. The prevalence and types of SDB varied in HF with different etiologies, which may be related to the different severities of HF. SDB was highly prevalent in patients with ischemic HF, hypertensive HF, and myocardial HF. Hypertensive HF patients were mainly complicated with OSA, and myocardial HF patients were mainly complicated with CSA. Both were highly prevalent in patients with ischemic HF, but OSA was higher. The prevalence of SDB was relatively low in patients with valvular and arrhythmic HF, and OSA was the main type.

## CONFLICTS OF INTEREST

The authors declare no conflicts of interest.

## Data Availability

The data used to support the findings of this study are available from the corresponding author upon request.
